# Simultaneous Catechol and Hydroquinone Detection with Laser Fabricated MOF-Derived Cu-CuO@C Composite Electrochemical Sensor

**DOI:** 10.3390/ma16227225

**Published:** 2023-11-18

**Authors:** Aleksandra Levshakova, Maria Kaneva, Evgenii Borisov, Maxim Panov, Alexandr Shmalko, Nikolai Nedelko, Andrey S. Mereshchenko, Mikhail Skripkin, Alina Manshina, Evgeniia Khairullina

**Affiliations:** 1 Institute of Chemistry, St. Petersburg State University, St. Petersburg 199034, Russia; sashkeens@gmail.com (A.L.); skt94@bk.ru (M.K.); or maksim.panov@pharminnotech.com (M.P.); st087489@student.spbu.ru (N.N.); a.mereshchenko@spbu.ru (A.S.M.); m.skripkin@spbu.ru (M.S.); 2 Ioffe Institute, St. Petersburg 194021, Russia; 3 Center for Optical and Laser Materials Research, St. Petersburg University, St. Petersburg 199034, Russia; eugene.borisov@spbu.ru; 4 Faculty of Pharmaceutical Technology, St. Petersburg State Chemical Pharmaceutical University, Professor Popov Str., 14, Lit. A, St. Petersburg 197022, Russia; 5 Nanotechnology Research and Education Centre RAS, Saint Petersburg Academic University, St. Petersburg 194021, Russia; sanya050199@gmail.com; 6 School of Physics and Engineering, ITMO University, St. Petersburg 191002, Russia

**Keywords:** flexible sensor, laser scribing, laser fabrication, hydroquinone, catechol, MOF-derived material

## Abstract

The conversion of metal-organic frameworks (MOFs) into advanced functional materials offers a promising route for producing unique nanomaterials. MOF-derived systems have the potential to overcome the drawbacks of MOFs, such as low electrical conductivity and poor structural stability, which have hindered their real-world applications in certain cases. In this study, laser scribing was used for pyrolysis of a Cu-based MOF ([Cu_4_{1,4-C_6_H_4_(COO)_2_}_3_(4,4′-bipy)_2_]_n_) to synthesize a Cu-CuO@C composite on the surface of a screen-printed electrode (SPE). Scanning electron microscopy, X-ray diffractometry, and Energy-dispersive X-ray spectroscopy were used for the investigation of the morphology and composition of the fabricated electrodes. The electrochemical properties of Cu-CuO@C/SPE were studied by cyclic voltammetry and differential pulse voltammetry. The proposed flexible electrochemical Cu-CuO@C/SPE sensor for the simultaneous detection of hydroquinone and catechol exhibited good sensitivity, broad linear range (1–500 μM), and low limits of detection (0.39 μM for HQ and 0.056 μM for CT).

## 1. Introduction

Metal-organic frameworks (MOFs) are a type of hybrid material that combines organic ligands and metal ions or clusters through coordination bonds. These unique structures have been extensively studied and have shown great potential as precursors for the synthesis of various nanomaterials [1,2]. By converting metal ions into metal or oxide particles and organic ligands into carbon-based matrices, MOFs can serve as parenting structures for advanced functional materials [3,4,5,6,7].

Various approaches for the pyrolysis of MOFs to produce MOF derivatives have been developed over the last decade. These methods can be broadly classified into two categories based on the rate of pyrolysis and the temperature ramp [8]. The first group involves a slow heating method, such as tube or muffle furnace thermal cracking [9]. On the other hand, the second category includes a rapid heating technique and is mostly related to laser-induced synthesis. The laser-assisted approaches offer significant advantages over conventional heating methods, such as fully automated manipulation, additive synthesis, high efficiency, and, most importantly, the achieving significantly higher temperatures than conventional approaches [10,11]. Due to extreme conditions provided by laser radiation, characteristics of the MOF-derived materials differ from those obtained through traditional pyrolysis methods and give rise to the MOF derivatives exhibiting unique physicochemical properties [8,12,13,14]. 

Moreover, laser-induced synthesis is less time-consuming in a range of orders of magnitude. The time-temperature profiles recorded during the synthesis process indicated a remarkably short duration of less than 2 s. In contrast, traditional pyrolysis methods required significantly longer processing times, typically spanning several hours [10].

In recent years, significant interest of researchers has been devoted to green catalysis, energy conversion, and environmental remediation. The distinct characteristics of laser-induced MOF derivatives make them promising candidates for multifunctional materials in these fields, which has been proven by many reports [15,16,17,18,19]. However, the full potential of laser-assisted synthesis in exploring MOF-derived materials for electrochemical sensor applications has not been fully realized. While there have been a limited number of studies on the fabrication of electrochemical sensors using laser pyrolysis of MOFs, this area remains promising for further research.

Among the electrochemically active substances, catechol (CT) and hydroquinone (HQ) are the ones with a high practical importance. These compounds find widespread use in the production of various goods, including leather, pharmaceuticals, dyes, and cosmetics [20]. Notably, CT and HQ are known for their high toxicity, resistance to degradation, and potential long-term adverse effects on human health [21]. Overexposure to these substances can lead to severe health issues such as kidney dysfunction, liver damage, fatigue, and others [22]. As a result of exposure to CT and HQ in the human body, many different reactions can occur with biomolecules such as DNA, proteins, and membranes, ultimately leading to permanent damage [23]. Prolonged exposure to such antioxidants in the human body leads to their accumulation since their absorption into the skin occurs faster than excretion in the urine [24]. The need for efficient and accurate quantification methods for HQ and CT is caused by possible groundwater and river pollution as a result of releasing these substances into the environment during manufacturing and usage [25].

This paper introduces a laser-induced technique for the synthesis of the MOF-derived Cu-CuO@C nanocomposite at ambient conditions. [Cu_4_{1,4-C_6_H_4_(COO)_2_}_3_(4,4′-bipy)_2_]_n_ was used as a precursor [26]. Copper and copper oxide NPs show excellent electrochemical activity toward various analytes, including HQ and CT [27,28,29,30,31]. Earth-abundant transition metals and metal oxides have excellent stability and decent performance in harsh environments such as strong acidic/alkaline solutions and high temperatures [32], as well as significant economic advantages. Among earth-abundant transition metals, copper stands out due to its lower toxicity (compared to Co) and higher corrosion resistance (in contrast to Fe), making copper and copper oxides promising for the fabrication of CT and HQ sensors. The addition of MWCNT allows one to benefit from the synergetic effect of different kinds of nanomaterials [33] and increase electrochemically active surface area, therefore enhancing the electrochemical properties of the fabricated sensor. Commercially available screen-printed electrodes (SPE) were used as conductive substrates for the accommodation of MOFs. The MOFs/MWCNT suspension on the surface of SPE was scribed with a CW 532 nm laser. The fabricated nanocomposite was characterized using scanning electron microscopy (SEM), X-ray diffractometry (XRD), and Energy-dispersive X-ray spectroscopy (EDX).

**Scheme 1:**
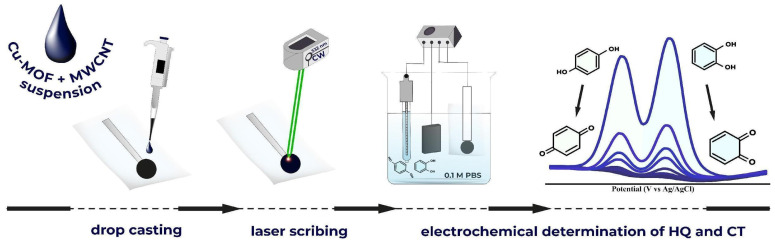
Electrode fabrication process and electrochemical testing.

The analytical performance of the sensor was evaluated by cyclic voltammetry (CV) and differential pulse voltammetry (DPV). The sensor demonstrated the ability for simultaneous detection of CT and HQ with good sensitivity, broad linear range, and low limits of detection. The pathway for fabrication of Cu-CuO@C/SPE is illustrated in Scheme 1.

## 2. Materials and Methods

### 2.1. Materials

Copper (II) nitrate trihydrate (Cu(NO_3_)_2_·3H_2_O), terephthalic acid (C_8_H_6_O_4_), 4,4′-bipyridine (C_10_H_8_N_2_), sodium dihydrogen phosphate dihydrate (NaH_2_PO_4_·2H_2_O), disodium hydrogen phosphate dodecahydrate (Na_2_HPO_4_·12H_2_O), potassium hexacyanoferrate(II) (K_4_[Fe(CN)_6_]), potassium chloride (KCl), catechol (C_6_H_6_O_2_) were purchased from Thermo Scientific (Fair Lawn, NJ, USA). Hydroquinone (C_6_H_6_O_2_) and Multi-walled carbon nanotubes (MWCNTs) were purchased from Macklin Biochemical Co, Ltd. (Shanghai, China). Isopropanol (C_3_H_7_OH, >99.8%) and ethanol (C_2_H_5_OH, 96%) were purchased from JSC LenReactiv (Saint Petersburg, Russia). All reagents were analytical grade and used without further purification. Ultrapure Milli-Q water was used for all experiments (18.2 MΩ cm).

### 2.2. Synthesis of [Cu_4_{1,4-C_6_H_4_(COO)_2_}_3_(4,4′-bipy)_2_]_n_

[Cu_4_{1,4-C_6_H_4_(COO)_2_}_3_(4,4′-bipy)_2_]_n_ was prepared according to the published procedure [26]. Copper nitrate trihydrate (0.600 g), 4,4′-bipyridine (0.388 g), and terephthalic acid (0.413 g) were dissolved in a water-ethanol solution (70% EtOH). The mixture was kept in an autoclave for 24 h at a temperature of 180 °C. Next, [Cu_4_{1,4-C_6_H_4_(COO)_2_}_3_(4,4′-bipy)_2_]_n_ samples were centrifuged and washed several times with ethanol and then dried in an oven overnight. The sample was assigned as Cu-MOF. The Cu-MOFs structure and XRD pattern with comparison to data from CSB are presented in Figure S1. 

### 2.3. Electrode Fabrication

The electrodes were prepared by using a Nd:YAG CW laser with a wavelength of 532 nm and a power output of up to 2 W. Commercially available screen-printed electrodes (Poten Inc., Qingdao, China) were used as a conductive substrate for accommodation of the MOFs-based suspension. The 5 μL solution of Cu-MOF (20 mg mL^−1^) and MWCNT (1 mg mL^−1^) dispersed in isopropanol were uniformly drop cast on the SPE. The modified SPE was left under ambient conditions until the complete evaporation of the alcohol. Thus, the electrode surface was scribed by a focused laser beam (NA 0.4). The laser power was varied within the range of 50 to 150 mW, while the scanning speed remained constant at 1.4 mm c^−1^. The writing process was performed using an XYZ-mechanical stage to control the movement of the substrate.

### 2.4. Characterization Techniques

X-ray diffraction (XRD) analysis was performed with a Bruker D2 Phaser diffractometer equipped with a LynxEye detector (Bruker-AXS, Karlsruhe, Germany) to establish the phase composition. Raman spectroscopy was carried out using a confocal spectrometer Senterra (Bruker, Billerica, MA, USA). The Raman spectra were performed by a 532 nm solid-state laser with 10 mW power using a 50× objective and were collected two times. The morphology and elemental analysis of the samples were investigated by scanning electron microscopy (SEM) on a Zeiss Merlin (Karl Zeiss, Oberkochen, Germany) equipped with a field emission cathode, a GEMINI-II electron-optics column, and an INCAx-act energy dispersive X-ray spectrometer (EDX) (Oxford Instruments, Abingdon, UK).

### 2.5. Electrochemical Measurements

The electrochemical experiments were carried out with a standard three-electrode cell using Corrtest CS300 potentiostat (Wuhan CorrTest Co. Ltd., Wuhan, China) at room temperature without Ar purging. The reference and counter electrodes were an Ag/AgCl (3 M KCl) and a platinum foil, respectively. Phosphate-buffered saline (0.1 M PBS) was used as a supporting electrolyte for the determination of HQ and CT. Cyclic voltammetry (CV) measurements were conducted within the potential window −0.1–0.8 V at a scan rate of 50 mV/s. The optimal DPV parameters were amplitude 0.05 V, inc. E 0.004 V, pulse width 0.05 s, pulse period 0.5 s. For the practical application test, local tap water was used as a real sample. The tap water was diluted with 0.1 M PBS in a ratio of 1:5 to prepare the sample for analysis. The standard-additions method was performed with 50 and 100 μM of HQ and CT, respectively.

## 3. Results

### 3.1. Preparation and Characterization of Cu-CuO@C/SPE via Laser Scribing

#### 3.1.1. Composition Optimization

In the proposed approach, according to the established mechanism, a laser is used to deliver energy to the metal ions in MOF crystals. This energy is transferred to the carbonaceous organic linkers, leading to the creation of reductive species. Subsequently, these reductive species reduce the metal ions into atoms, which are gathered to form NPs [11]. These resulting nanoparticles are dispersed within the porous carbon derived from the ligands, which provides long-term stability to the nanoparticles [34]. It has been observed that the laser treatment in a nitrogen environment transforms the metal ions into metal nanoparticles; however, oxidation may occur during the synthesis under an ambient atmosphere. 

The structural transformation of MOF during the laser synthesis was comprehensively studied by means of various techniques, including SEM, EDX, XRD, and Raman spectroscopy. 

**Figure 1:**
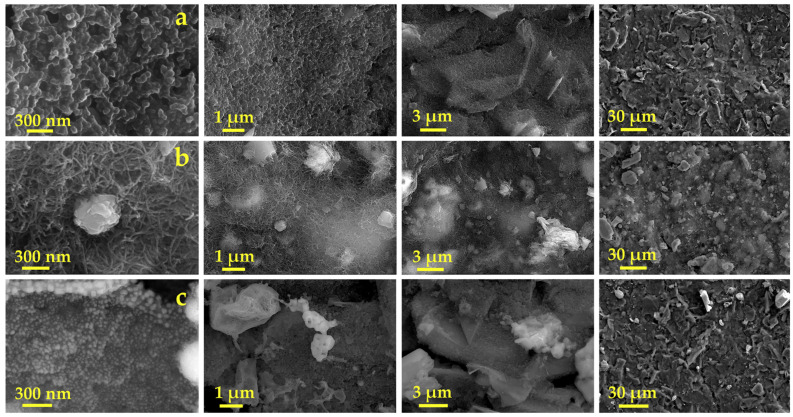
SEM images of samples at different magnifications scribed SPE (row **a**), Cu-MOF/MWCNT/SPE (row **b**), and Cu-CuO/SPE (row **c**).

For the proper design of the material, the interaction of the laser beam with individual system components was studied; this includes scribed blank SPE, Cu-CuO/SPE, and Cu-MOF/MWCNT/SPE without laser treatment. The corresponding SEM images are presented in Figure 1. It was observed that the laser-scribed blank SPE does not exhibit any visible morphological changes compared to the as-received electrode (Figure 1a and Figure S2). The Cu-MOF/MWCNT/SPE without laser treatment displays a satisfactory morphology, with the Cu-MOF and MWCNT well dispersed and evenly distributed over the working part of the SPE, providing the necessary background for further laser treatment (Figure 1b). In turn, the laser irradiation of Cu-MOF results in the destruction of the frameworks and the formation of nanoparticles (Figure 1c). 

The Raman data support and complement the observation made by SEM. As-received SPE reveals peaks specific to partially oxidized highly oriented graphite (Figure S3a). After laser treatment, amorphization occurs; this can be traced by the change in the relative intensity of the D and G peaks (Figure S3b) [35]. Similarly, the same changes occur for the laser-scribed Cu-CuO/SPE. The carbon amorphization can be attributed to the transformation of MOF ligands and the amorphization of the SPE after laser scribing (Figure S3c). Moreover, peaks corresponding to the CuO bands appear, confirming the Cu-MOF decomposition [36]. After Cu-MOFs/MWCNT suspension drop casting, Raman spectra reveal the peaks corresponding to MWCNT (Figure S3d) [37]. 

XRD measurements were performed for the identification of phases present in the materials. All patterns show reflexes corresponding to graphite and graphite oxide, which originate from the SPE [38]. In the case of Cu-MOF/MWCNT/SPE, weak peaks of Cu-MOF and MWCNT were observed [39]. However, no significant changes in the XRD patterns were observed for the as-received SPE, scribed SPE, and Cu-CuO/SPE (Figure S5; this could be attributed to the low amount of precursors deposited on the surface of the SPE, resulting in a low amount of newly synthesized phases.

#### 3.1.2. Laser Scribing Optimization

**Figure 2:**
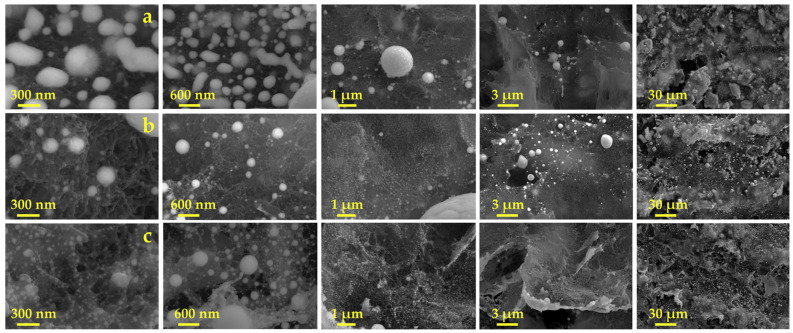
SEM images Cu-CuO@C/SPE synthesized at different laser powers: 50 mW (row **a**), 100 mW (row **b**), 150 mW (row **c**).

The different laser treatment conditions were investigated to study the structural evolution of MOF/MWCNT and to develop electrode material with notable performance as an electrochemical sensor for HQ and CT. SEM images of Cu-CuO@C/SPE after the action of laser radiation with different power (50 mW, 100 mW, 150 mW) show the nanoparticles with wide size distribution (Figure 2).

The formation of particles with different sizes can be attributed to the interplay between the reduction rate of copper ions and the consumption rate of copper clusters during particle growth. 

When the reduction rate surpasses the consumption rate, the concentration of reduced copper atoms tends to remain above a critical supersaturation level for a longer period of time. This prolonged state allows for an extended nucleation period or multiple nucleation events, resulting in nuclei with differing growth periods. Consequently, the final particles exhibit a broader range of sizes compared to those grown at a constant rate following a single nucleation event [40]. 

The tendency for the formation of smaller particles under the influence of higher energies can be explained by considering the nucleation rate (k_1_) and growth rate (k_2_) constants. This theory was experimentally proven at significantly lower temperatures than reached in the case of laser-assisted processes [41]; nevertheless, it is possible to assume that processes after cooling have the same nature and lead to similar results. In [41], authors describe two regimes of NP formation: one with a sufficient amount of metal ions and the other with an insufficient amount, such as an excess of reducing agents. Highly likely, our experimental conditions fall within the second regime. Given the composition of Cu-MOF, it is possible for multiple molecules of reducing gases (such as CO and H_2_) to form per copper atom [8]. In this scenario, an increase in temperature would lead to an increase in k_1_, resulting in explosive nucleation that consumes a large quantity of metal ions. As a result, growth would be limited due to a lack of Cu^2+^ precursors.

**Figure 3:**
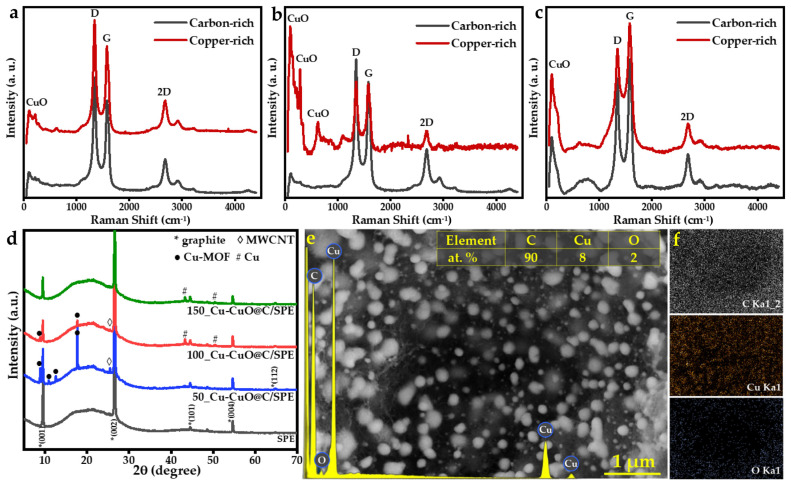
Raman scattering spectra of electrodes (**a**) 50_CuCuO@C/SPE, (**b**) 100_CuCuO@C/SPE, (**c**) 150_CuCuO@C/SPE. Red lines correspond to copper-rich parts of the samples, and gray lines correspond to carbon-rich parts. (**d**) XRD pattern SPE, 50_CuCuO@C/SPE, 100_CuCuO@C/SPE, 150_Cu-CuO@C/SPE. (**e**) SEM + EDX of 100_CuCuO@C/SPE, (**f**) distribution of elements on the surface of 100_Cu-CuO@C/SPE.

The morphology of the samples treated at various power levels differs significantly. In the sample treated with lower energy, there is a presence of unconverted and partially converted MOF crystals (Figure S4). Additionally, Figure 2c shows changes in the morphology of MWCNT after laser treatment with a power of 150 mW. The Raman spectroscopy supports these findings and shows that carbon amorphization occurs at a high power of laser radiation (Figure 3a–c). Considering the extremely high thermal stability of MWCNT up to 2200 °C [42,43], it is highly unlikely to reach such extreme temperatures under the given experimental conditions. Such temperatures would lead to the distraction of SPE and PET polymer substrate [44]. Another factor to consider is the plasmonic heating effect on the growing copper NPs. Copper NPs have a wide absorption spectrum with a maximum lying in the range of 560–580 nm [45,46,47], meaning that the 532 nm radiation used in our experiments is not resonant with the plasmon and does not lead to effective plasmon heating. Furthermore, it was shown that the excitation of CuNWs with a power of 937 mW at 808 nm only increases the temperature to 200 °C within 2 s [48]. Based on these considerations, it is reasonable to suggest that the layer of amorphous carbon phase originates from MOF linkers. However, further thorough investigation is needed in the future to confirm this hypothesis, as it exceeds the scope of the present article.

Raman spectra were collected from two specific regions of the sample using a microscope, targeting the carbon-rich and copper-rich areas (Figure 3a–c). 

Analysis of the spectra revealed that at the lower and medium laser powers (50 and 100 mW), the intensity of the D peak exceeded that of the G peak (Figure 3a,b), unlike the sample treated with 150 mW (Figure 3c); this implies that laser powers of 50 and 100 mW are effective in maintaining a highly oriented carbon structure [49]. It is important to mention the existence of a minor peak with low intensity around 2450 cm^−1^, indicating the presence of the oxidized carbon. Moreover, the Raman signals related to CuO are more prominent at 100 and 150 mW (Figure 3b,c) [50,51].

However, the XRD analysis does not indicate the presence of copper oxides. The XRD patterns show a gradual emergence of copper reflections [52,53] while the Cu-MOF reflections disappeared, accompanied by the presence of graphite reflections from SPE (Figure 3d). Furthermore, the EDX mapping confirmed that copper was evenly distributed throughout the sample, consistent with the SEM image and the placement of nanoparticles. Elemental analysis revealed an atomic percentage ratio of Cu to O of 4:1 (Figure 3e,f). Part of these oxygen atoms is bound to carbon; thus, the amount of O bound to copper is even less. Based on these findings, it can be concluded that the laser scribing process leads to the formation of copper nanoparticles. Moreover, partial copper oxidation may occur during fabrication and can be a result of post-synthesis storage processes in an air environment.

### 3.2. Electrochemical Properties of Cu-CuO@C/SPE

The electrochemical behavior of the fabricated electrodes was investigated by CV measurements (Figure 4a,b) in supporting electrolytes (0.1 PBS) and in the presence of 100 µM of HQ and 100 µM of CT. The anodic peaks in the positive-going scan and their corresponding cathodic peaks in the negative-going scan represent the transformation of catechol to 1,2-benzoquinone and hydroquinone to 1,4-benzoquinone (Figure S6).

**Figure 4:**
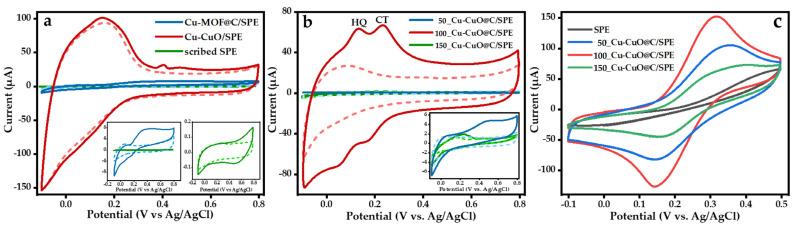
(**a**) CVs of electrodes, treated with different modifications, recorded in background 0.1 M PBS (dashed line) and presence of 100 µM of HQ and 100 µM of CT (solid line); (**b**) CVs of electrodes, fabricated at different laser powers, recorded in background 0.1 M PBS (dashed line) and presence of 100 µM of HQ and 100 µM of CT (solid line); (**c**) CVs recorded in 5.0 mM [Fe(CN)_6_]^3−/4−^ solution containing 0.1 M KCl.

Modification by suspension with Cu-MOF and MWCNT without laser treatment gives only a slight improvement compared to as-received SPE. In turn, scribing of Cu-MOF on the SPE surface leads to an increase in CV response. Despite this, the oxidation peaks of HQ and CT are not well pronounced (Figure 4a). To address this issue, MWCNTs were added to increase the electrochemical surface area and facilitate the absorption of organic analytes. The influence of laser power on the CV response was studied to determine the optimal conditions for enhancing the electrode’s electrochemical activity (Figure 4b). The formation of amorphous carbon at higher energies results in poor electrochemical response, presumably due to lower surface area and number of available active sites. On the other hand, lower laser power is not sufficient for full Cu-MOF pyrolysis. Therefore, the optimum laser energy is determined to be in the middle range, achieving a balance between carbon amorphization and copper NP formation. The electron transfer behavior of the samples in a 5.0 mM [Fe(CN)_6_]^3−/4−^ exhibited the same trend (Figure 4c). The electrode fabricated at 100 mW displayed higher currents and a smaller peak separation, indicating the most favorable electrochemical performance among the tested electrodes. Based on the comprehensive characterizations performed, the Cu-CuO@C/SPE electrode fabricated at 100 mW was selected for the electrochemical determination of CT and HQ.

### 3.3. Electrochemical Determination of Hydroquinone and Catechol

The determination of HQ and CT was performed using differential pulse voltammetry (DPV). The pH optimization of supporting solutions (pH = 3, 5, and 7) containing 100 μM HQ and CT was performed (Figure S7). Evaluation of solutions with pH above 7 was not meaningful due to low proton availability and the instability of HQ and CT at high pH [54]. The current intensity significantly depends on the pH of the solution, influenced by various factors. At low pH values, the protonation of the hydroxyl groups of catechol and hydroquinone leads to reduced adsorption on the electrode surface [55]. Furthermore, a shift to higher potentials with increasing pH was observed [56]. According to these findings, further investigations were performed at pH = 7.

**Figure 5:**
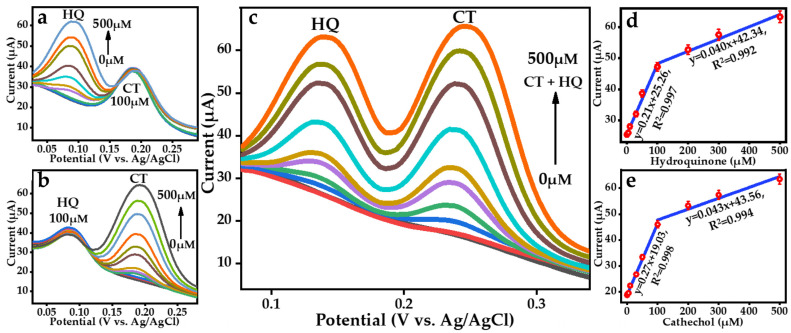
(**a**) DPVs recorded in 0.1 M PBS at a fixed concentration of 0.1 mM CT and different HQ concentrations (1 to 500 µM); (**b**) DPVs recorded in 0.1 M PBS at a fixed concentration of 0.1 mM HQ and different CT concentrations (0.1 to 500 µM); (**c**) DPV illustrating the effect of simultaneous addition of different concentrations of CT and HQ (from 0.1 to 500 µM) in 0.1 M PBS; (**d**) calibration curve between different concentrations of HQ vs. anodic peak; (**e**) calibration curve between different concentrations of CT vs. anodic peak.

The two types of the model systems were studied: (1) fixed concentration of one analyte at variation of another (Figure 5a,b and Figure S8), (2) simultaneous variation of concentration for both substances (Figure 5c–e). The Cu-CuO@C/SPE electrode demonstrates separation of HQ and CT peaks by 107 mV. In both experimental designs, the linear range of the sensor is divided into two regions: 0 to 100 μM and 100 to 500 μM for HQ and CT. Typically, the first linear region shows a rapid increase in response with the concentration of analyte, indicating high sensitivity. The second linear region often demonstrates lower sensitivity, attributed to the adsorption of intermediates at high concentrations of analyte [57,58,59]. The linear regression equation for the simultaneous variation of concentration is presented in Figure 5d,e. The calibration curves for model system 1 (Figure S8) show good agreement with Figure 5c; they demonstrate similar linear ranges and sensitivity.

The limit of detection (LOD) for HQ is 0.39 μM, while for CT, it is 0.056 μM (S/N = 3). This sensor exhibits superior or comparable characteristics to other reported works, including well-established systems based on modified glassy carbon electrodes (Table S1). The detection limits are found to be 0.39 and 0.056 μM (S/N = 3) for HQ and CT, respectively. The performance of the sensor developed in this study is comparable to previously reported works (see Table S1); however, Cu-CuO@C/SPE has strong advantages over similar electrodes, such as simple fabrication procedure and flexibility.

To test the selectivity of the Cu-CuO@C/SPE electrode, the impact of the interference substance on the determination of 50 μM HQ and 50 μM CT was investigated. The signal changes observed for HQ and CT were equal to less than 4.0% after the addition of K^+^, Na^+^, Fe^3+^, Mg^2+^, Cl^−^, NO^3−^, SO_4_^2−^, PO_4_^3−^, phenol, and bisphenol A to the supporting electrolyte (Table S2). These results prove the electrodes have high interference resistance to the most abundant coexisting species. Reproducibility experiments were conducted using three electrodes, and RSDs of the peak current did not exceed 4.2%. This good reproducibility, along with other outstanding characteristics, can be attributed not only to the composition of the electrode material but also to the enhanced adhesion of the Cu-CuO@C composite to the SPE surface compared to the untreated suspension (Figure S9). The laser scribing results in the formation of an electrocatalytically active material that is strongly embedded into the surface of the SPE.

The practical value of the Cu-CuO@C/SPE sensor was confirmed by conducting the measurements with real samples of tap water using the standard addition method. The RSD was found to be less than 3.4% for both analytes, while the calculated recoveries lie within a range of 98.2–102.2% and 97.2–99.5% for HQ and CT, respectively (Table S3). Real samples application completes the impressive analytical parameters of the Cu-CuO@C/SPE sensor for the detection of HQ and CT.

## 4. Conclusions

In this paper, a laser-assisted technique for the synthesis of the MOF-derived electrode material at ambient conditions has been proposed. Commercially available screen-printed electrodes (SPE) were used as conductive substrates for the accommodation of the MOF-derived nanocomposite. To enhance the electrochemical properties of the fabricated sensor, multi-walled carbon nanotubes (MWCNT) were added to benefit from the synergistic effect of different nanomaterials and increase the electrochemically active surface area. Cu-CuO@C/SPE laser-scribed electrochemical sensors for HQ and CT were successfully developed. A detailed analysis of the Cu-CuO@C/SPE electrochemical properties demonstrates impressive analytical performance with low LODs (0.056 and 0.39 μM for CT and HQ, respectively) and a wide range of detectable concentration (1–500 μM for both substances). Furthermore, the flexible Cu-CuO@C/SPE electrode has excellent interference resistance and reproducibility, thereby showing great potential for practical application.

## Data Availability

Data available on request.

## Supplementary Materials

The following supporting information can be downloaded at https://www.mdpi.com/article/10.3390/ma16227225/s1. Figure S1. (a) Structure of [Cu_4_{1,4-C_6_H_4_(COO)_2_}_3_(4,4′-bipy)_2_]_n_ (Cu-MOF) (Cu—orange, O—red, N—blue, C—grey); (b) XRD pattern of (Cu-MOF) and CSD 147561. Figure S2. SEM images of as-received SPE at different approximations. Figure S3. Raman scattering spectra of electrodes, treated with different modifications (a) SPE, (b) scribed SPE, (c) 100_Cu-CuO/SPE, (d) Cu-MOF/MCWNT/SPE. Figure S4. SEM images of partially converted Cu-MOF at different magnifications. Figure S5. XRD pattern SPE, scribed SPE, Cu-MOF/MWCNT/SPE. Figure S6. Electrooxidation mechanism of CT (a) and HQ (b). Figure S7. DPV of Cu-CuO@C/SPE electrode recorded in 0.1 M PBS and 0.1 M PBS containing 100 μM HQ and 100 μM CT, at pH = 3, 5, 7. The graph in the inset shows the linear relationship between pH and analytical signal. Figure S8. Calibration curve between different concentrations of CT in the presence of 100 μM HQ vs. anodic peak; (e) Calibration curve between different concentrations of HQ in the presence of 100 μM CT vs. anodic peak. Figure S9. Peel test before and after laser scribing. Table S1. Performance comparison of 100_Cu-CuO@C/SPE for CT and HQ with other sensors [60,61,62,63,64,65,66,67,68,69]. Table S2. Influence of interference agents on the determination of 50 μM HQ and 50 μM CT. Table S3. Determination of HQ and CT in tap water samples.
