# Supplementary material for: Simultaneous Catechol and Hydroquinone Detection with Laser Fabricated MOF-Derived Cu-CuO@C Composite Electrochemical Sensor
Source: Materials (Basel). 2023 Nov 18;16(22):7225. doi: 10.3390/ma16227225 (PMC10673110; doi:10.3390/ma16227225)
Supplement: Supplementary file 1 [file materials-16-07225-s001.zip › materials-2699544-supplementary.pdf]

materials

Supplementary Materials

Simultaneous Catechol and Hydroquinone Detection with Laser Fabricated MOF-Derived Cu-CuO@C Composite Electrochemical Sensor

Aleksandra Levshakova [1], Maria Kaneva [1,2], Evgenii Borisov [3], Maxim Panov [1,4,5], Alexandr Shmalko [5], Nikolai Nedelko [1], Andrey S. Mereshchenko [1], Mikhail Skripkin [1], Alina Manshina [1,*] and Evgeniia Khairullina [1,6,*]

[1] Institute of Chemistry, St. Petersburg State University, St. Petersburg 199034, Russia; sashkeens@gmail.com (A.L.); skt94@bk.ru (M.K.); m.s.panov@spbu.ru or maksim.panov@pharminnotech.com (M.P.); st087489@student.spbu.ru (N.N.); a.mereshchenko@spbu.ru (A.S.M.); m.skripkin@spbu.ru (M.S.)
[2] Ioffe Institute, St. Petersburg 194021, Russia
[3] Center for Optical and Laser Materials Research, St. Petersburg University, St. Petersburg 199034, Russia; eugene.borisov@spbu.ru
[4] Faculty of Pharmaceutical Technology, St. Petersburg State Chemical Pharmaceutical University, Professor Popov Str., 14, Lit. A, St. Petersburg 197022, Russia
[5] Nanotechnology Research and Education Centre RAS, Saint Petersburg Academic University, St. Petersburg 194021, Russia; sanya050199@gmail.com
[6] School of Physics and Engineering, ITMO University, St. Petersburg 191002, Russia
* Correspondence: a.manshina@spbu.ru (A.M.); e.khayrullina@spbu.ru (E.K.)

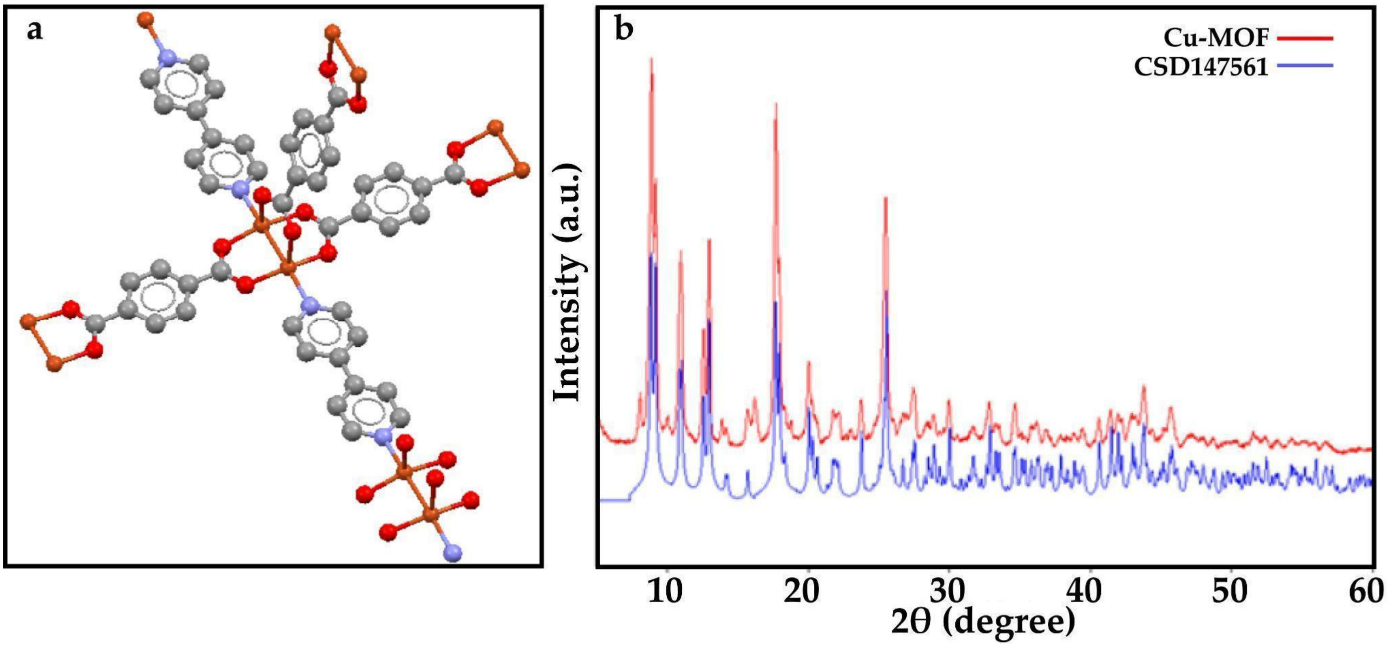

Figure S1. (a) Structure of $[Cu_4\{1,4\text{-}C_6H_4(COO)_2\}_3(4,4'\text{-bipy})_2]_n$ (Cu-MOF) (Cu – orange, O – red, N – blue, C - grey); (b) XRD pattern of (Cu-MOF) and CSD 147561.

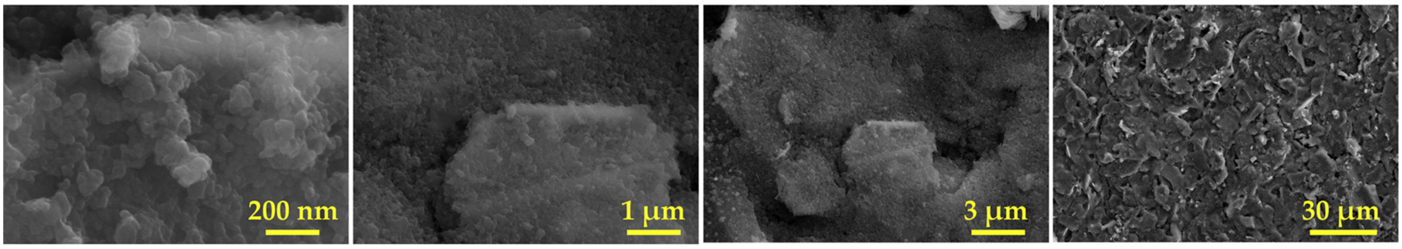

Figure S2. SEM images of as-received SPE at different approximation.

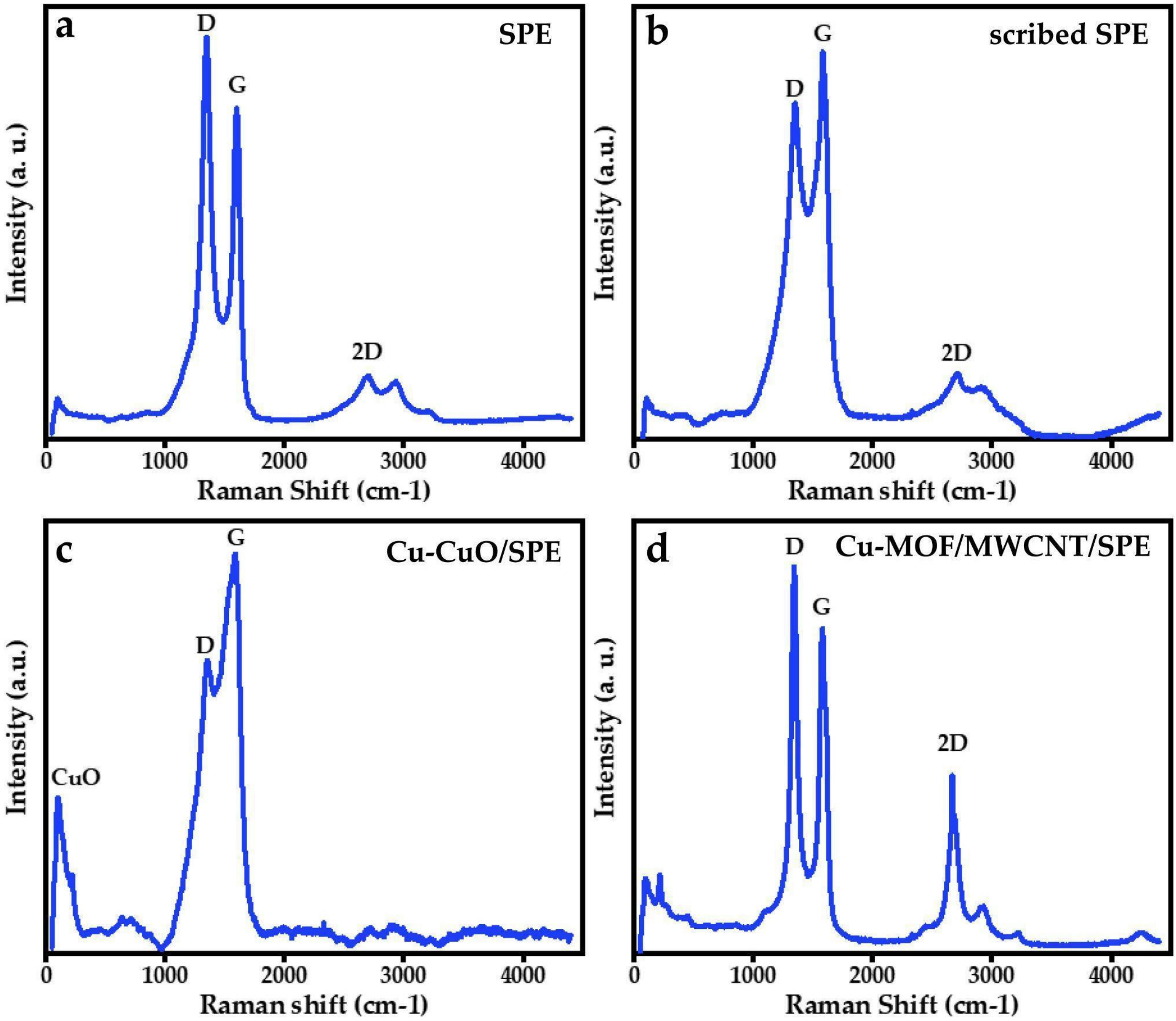

Figure S3. Raman scattering spectra of electrodes, treated with different modifications (a) SPE, (b) scribed SPE, (c) 100_Cu-CuO/SPE, (d) Cu-MOF/MCWNT/SPE.

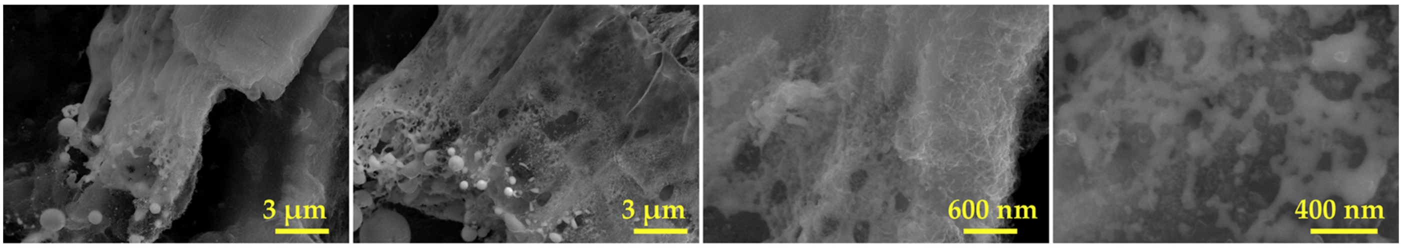

Figure S4. SEM images of partially converted Cu-MOF at different magnifications.

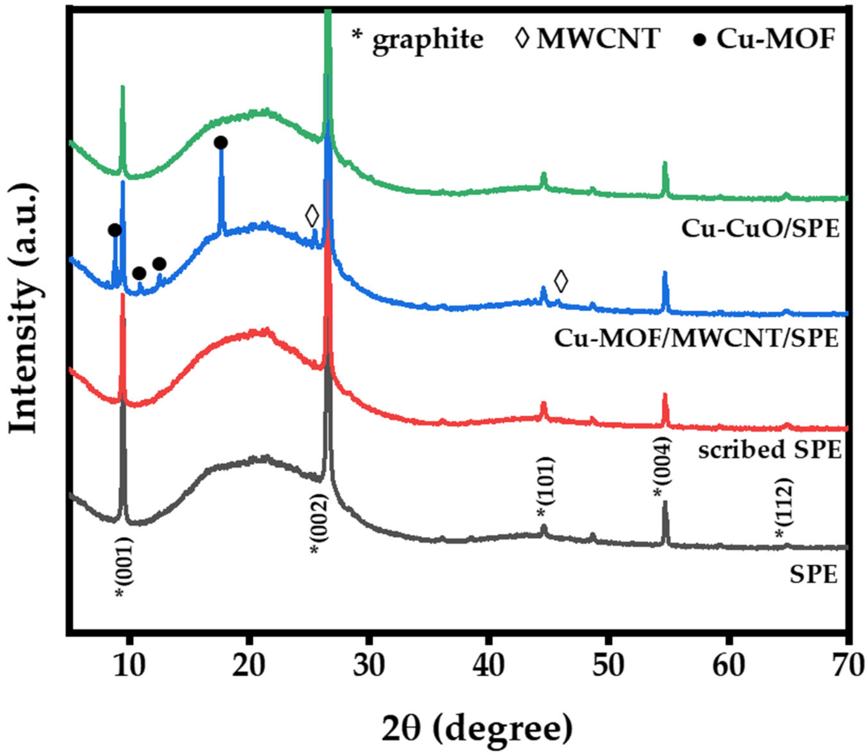

Figure S5. XRD pattern SPE, scribed SPE, Cu-MOF/MWCNT/SPE

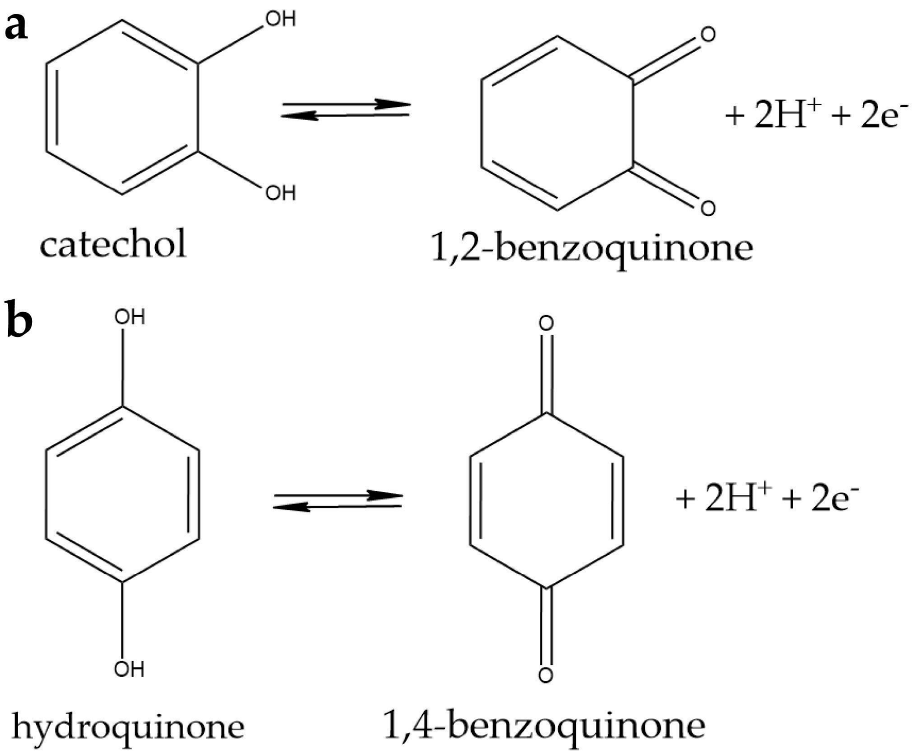

Figure S6. Electrooxidation mechanism of CT (a) and HQ (b).

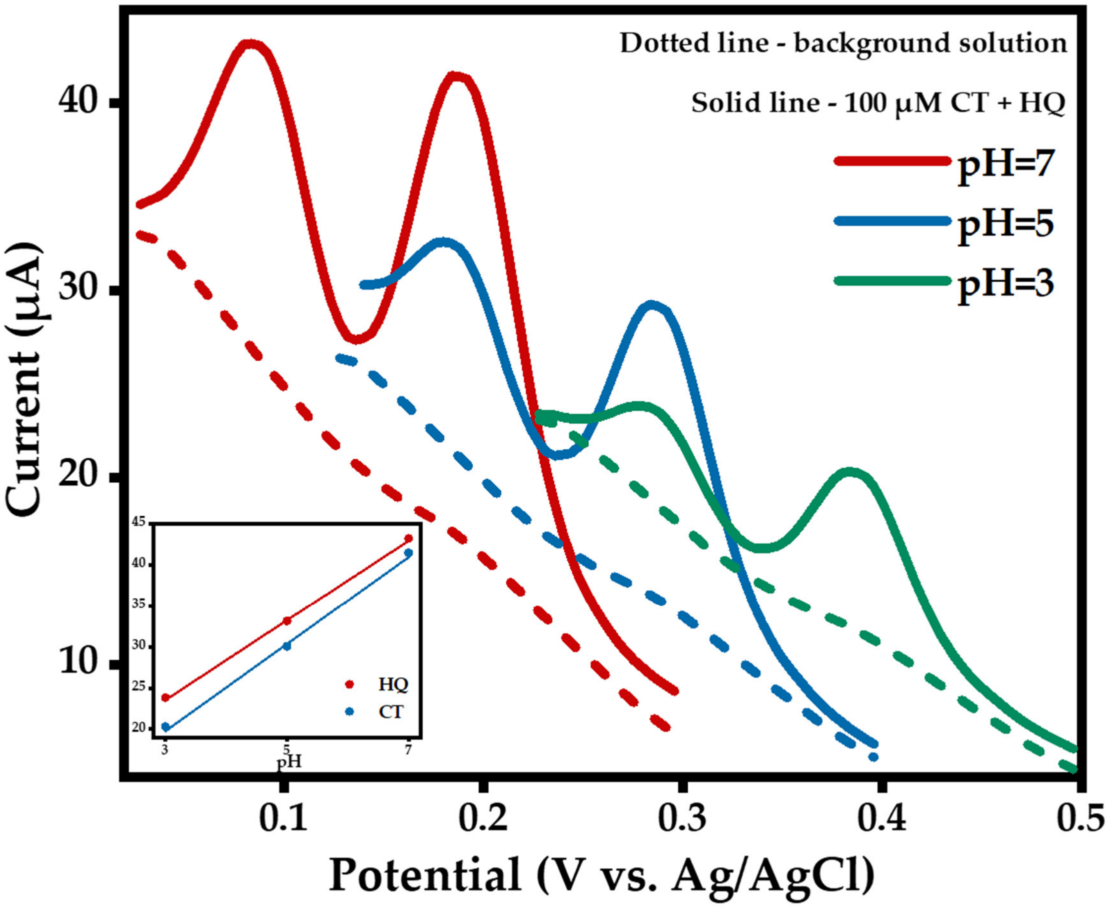

Figure S7. DPV of Cu-CuO@C/SPE electrode recorded in 0.1 M PBS and 0.1 M PBS containing 100 μM HQ and 100 μM CT, at pH=3, 5, 7. The graph in the inset shows the linear relationship between pH and analytical signal.

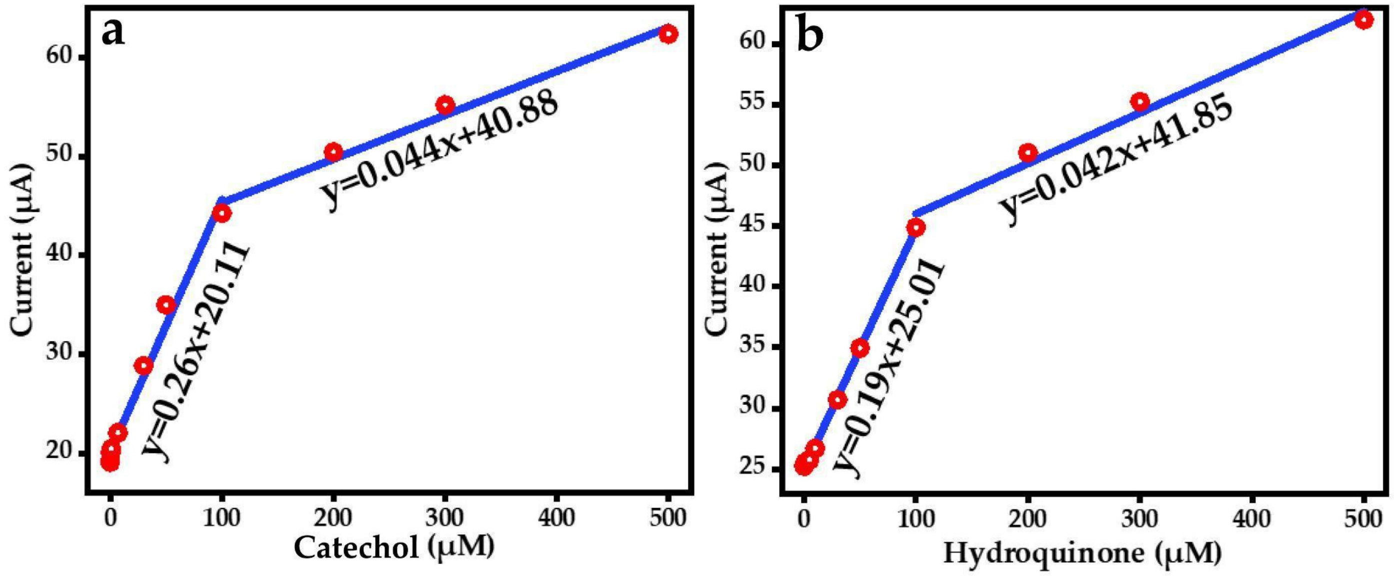

Figure S8. Calibration curve between different concentrations of CT in presence of 100 μM HQ vs. anodic peak; Calibration curve between different concentrations of HQ in presence of 100 μM CT vs. anodic peak.

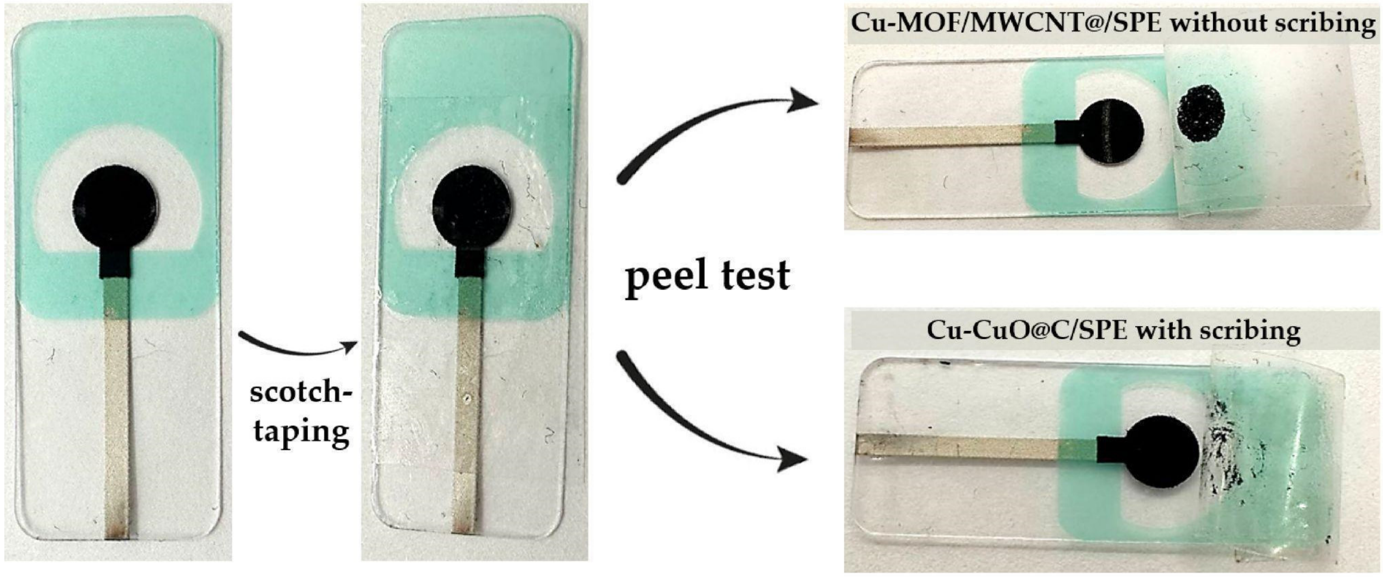

Figure S9. Peel test before and after laser scribing.

.

Table S1. Performance comparison of 100_Cu-CuO@C/SPE for CT and HQ with other sensors.

Electrode material	Technique	Electrolyte	Range of HQ (μM)	Range of CT (μM)	LOD of HQ (μM)	LOD of CT (μM)	Ref.
CNF-CuO/GCE	DPV	ABS pH 7.1	3–80	0–150	1.0	2.0	[60]
Cu-CuO@MoOx-PEDOT/PET	CV	PBS pH 7.4	5–870	5–885	0.221	0.216	[61]
GN-Cu-MOF/GCE	DPV	PBS pH 7.0	1–1000	1–1000	0.590	0.330	[62]
MWCNTs/CDs/MWCNTs/GCE	DPV	PBS pH 7.0	1–200	4–200	0.070	0.060	[63]
ERGO-MOF/GCE	DPV	PBS pH 6.0	0.1–476	0.1–566	0.1	0.1	[64]
GQD-CuO-His/GCE	DPV	PBS pH 7.0	0.001–40	-	0.31	-	[65]
PPGE	DPV	PBS pH 7.0	2–200	2–200	1.17	1.32	[66]
PEDOT/DGNs/GCE	CV	PBS pH 7.0	5–200	5–100	1.4	0.9	[67]
Poly(Evansblue)/carbon pasteeletrode	CV	PBS pH 7.4	10–45	10–40	2.1	2.0	[68]
Zn/CuO Npcs	DPV	PBS pH 7.4	10–90	10–90	7	6	[69]
100_Cu-CuO@C/SPE	DPV	PBS pH=7.0	1–100<br>100–500	0.1–100<br>100–500	0.39	0.056	This work

Table S2. Influence of interference agents on the determination of 50 μM HQ and 50 μM CT.

Interfering agent	Fold excess concentration	Signal change (%)
K^+	30	1.5
Na^+	30	2.8
Fe^{3+}	30	3.9
Mg^{2+}	30	2.6
Cl^-	30	4.0
NO^{3-}	30	2.7
SO_4^{2-}	30	2.7
PO_4^{3-}	30	1.4
Bisphenol A	1	4.1
Phenol	1	4

Table S3. Determination of HQ and CT in tap water samples.

**Sample No**	**HQ**				**CC**			
	**Added,** μM	**Found,** μM	**Recovery,** %	**RSD[a] ,** %	**Added,** μM	**Found,** μM	**Recovery,** %	**RSD[a] ,** %
1	50	51.1	102.2	3.4	50	48.6	97.2	2.1
2	100	98.2	98.2	2.8	100	99.5	99.5	1.8

[a]*Measurement values taken from three experiments*
